# Comparative Genomics of *Mycobacterium avium* Subspecies *Paratuberculosis* Sheep Strains

**DOI:** 10.3389/fvets.2021.637637

**Published:** 2021-02-15

**Authors:** Rachel Mizzi, Verlaine J. Timms, Marian L. Price-Carter, Milan Gautam, Richard Whittington, Cord Heuer, Patrick J. Biggs, Karren M. Plain

**Affiliations:** ^1^Farm Animal Health Group, Sydney School of Veterinary Science, Faculty of Science, The University of Sydney, Camden, NSW, Australia; ^2^Centre for Infectious Diseases and Microbiology, Public Health, Westmead Hospital, Westmead, NSW, Australia; ^3^AgResearch, Hopkirk Research Institute, Palmerston North, New Zealand; ^4^School of Veterinary Science, Massey University, Palmerston North, New Zealand; ^5^School of Fundamental Sciences, Massey University, Palmerston North, New Zealand

**Keywords:** Johne's disease, *Mycobacterium avium* subspecies *paratuberculosis*, pan-genome, sheep strain, whole genome sequencing, type I, type III

## Abstract

*Mycobacterium avium* subspecies *paratuberculosis* (MAP) is the aetiological agent of Johne's disease (JD), a chronic enteritis that causes major losses to the global livestock industry. Further, it has been associated with human Crohn's disease. Several strains of MAP have been identified, the two major groups being sheep strain MAP, which includes the Type I and Type III sub-lineages, and the cattle strain or Type II MAP lineage, of which bison strains are a sub-grouping. Major genotypic, phenotypic and pathogenic variations have been identified in prior comparisons, but the research has predominately focused on cattle strains of MAP. In countries where the sheep industries are more prevalent, however, such as Australia and New Zealand, ovine JD is a substantial burden. An information gap exists regarding the genomic differences between sheep strain sub-lineages and the relevance of Type I and Type III MAP in terms of epidemiology and/or pathogenicity. We therefore investigated sheep MAP isolates from Australia and New Zealand using whole genome sequencing. For additional context, sheep MAP genome datasets were downloaded from the Sequence Read Archive and GenBank. The final dataset contained 18 Type III and 16 Type I isolates and the K10 cattle strain MAP reference genome. Using a pan-genome approach, an updated global phylogeny for sheep MAP from *de novo* assemblies was produced. When rooted with the K10 cattle reference strain, two distinct clades representing the lineages were apparent. The Australian and New Zealand isolates formed a distinct sub-clade within the type I lineage, while the European type I isolates formed another less closely related group. Within the type III lineage, isolates appeared more genetically diverse and were from a greater number of continents. Querying of the pan-genome and verification using BLAST analysis revealed lineage-specific variations (*n* = 13) including genes responsible for metabolism and stress responses. The genetic differences identified may represent important epidemiological and virulence traits specific to sheep MAP. This knowledge will potentially contribute to improved vaccine development and control measures for these strains.

## Introduction

Johne's Disease (JD) is a chronic gastroenteritis of ruminant species worldwide (1). Additionally, the causative agent, *Mycobacterium avium* subspecies *paratuberculosis* (MAP), has been implicated in the pathobiology of Crohn's disease in humans. Despite a strong association, causality has not been proven (2, 3). A major complication of JD is the extended subclinical phase. During this stage, low levels of intermittent bacterial shedding occurs in the faeces. Low levels of shedding may be difficult to detect with current diagnostic tests and may lead to false negative results. This insufficient sensitivity may allow for ongoing spread of the pathogen (4). Overall, losses due to clinical disease tend to be highest in dairy cattle since animals are retained in herds to older ages and hence have more time to develop disease. An Australian study estimated an average cost of $2,491AUD per cow with clinical JD (5). A similar value of $2,386AUD per clinical case was estimated by a French study (6). In sheep, the main losses are attributed to mortalities which have been estimated at 1–10% in Australian flocks (1, 7). Stud operations may become unviable if JD is prevalent due to restrictions imposed on sales and a reduced customer base (8). Thus, control of MAP and the ability to trace the spread of disease is critical. Further studies are required to inform producers of the economic losses and the cost-benefit for JD control measures in sheep enterprises.

**Figure 1:**
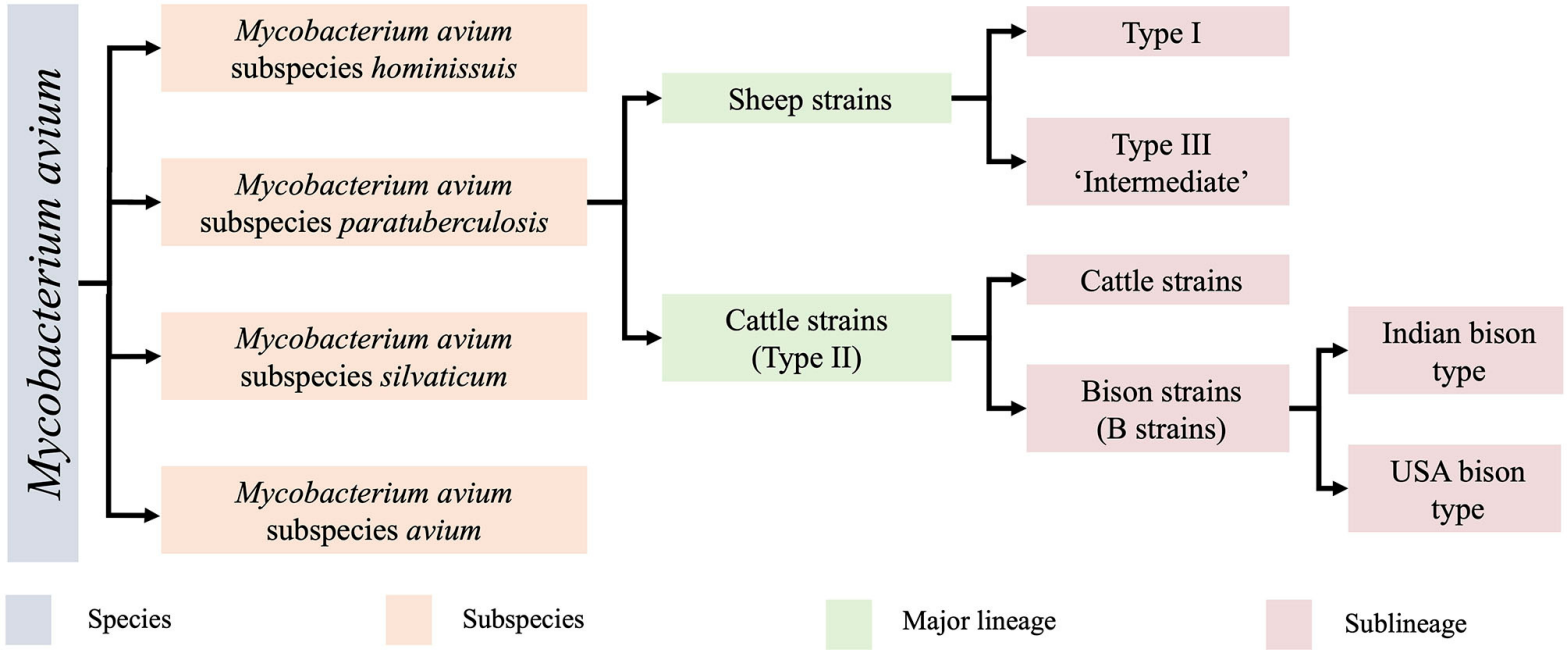
The *Mycobacterium avium* species and lineages of MAP and their associated nomenclature. Originally identified by low resolution techniques, clarification of these lineages has now been demarcated by whole genome sequencing (9, 10). The Type I and Type III sheep sublineages are the focus of the present study.

MAP is one of four closely related subspecies within the species *Mycobacterium avium* (Figure 1). Within this subspecies, two major groups are recognised, the sheep strains (S strains) and cattle strains (C strains), which were named after the host from which they were originally isolated (11). S and C strains of MAP can be distinguished by a variety of molecular methods including variation in the *IS*1311 sequence (12), variable number tandem repeats (VNTR) and short sequence repeat (SSR) loci (13, 14). Typing methods have helped to determine that MAP strains are cross transmissible between ruminant species (14–16), leading to some researchers preferring to designate S strains as Type I and C strain as Type II to avoid confusion. Within the two major groups, several sub-lineages of MAP strains exist. The C strain/Type II lineage contains a subcategory of bison strains, which were originally thought to be their own lineage (17, 18) but were recently demonstrated to be a sub-lineage of Type II by whole genome sequencing (WGS) (9) with regional lineages present in India and America (18). Within the S group there are two sub-lineages, Type I and Type III. The Type III strains were initially thought to be an intermediate of sheep and cattle strains (11, 19) but were later proven to be a sub-lineage of S strains by WGS (9) (Figure 1).

Accurate strain identification of MAP is vital for source attribution and mapping transmission pathways in epidemiological investigations. Furthermore, it improves the knowledge of bacterial population structure. This betters our understanding of the genetic diversity that exists in a population and potentially links a genotype with a disease outcome, transmission risk factors or origin of infection in the case of a new outbreak if the transmission chain is unknown. Specific control strategies may be warranted in the case of a new outbreak if the transmission chain is known. This is particularly important for a pathogen such as MAP which has multiple host species, and therefore different potential agro-industrial or wildlife sources and may be zoonotic. Historically, strain typing required culture of MAP, a process that is very time-consuming and may result in false negative results (20) if an inappropriate culture medium is used. This is particularly important for sheep strains, which are notoriously difficult to culture; typing was originally undertaken after physically extracting bacterial cells from intestinal mucosa (21). Phenotypic differences between S and C strains have been described that relate to culture requirements and virulence (22), with S strains appearing less virulent *in vivo* in terms of the ability to infect multiple species and also *in vitro* in models when human or bovine macrophages are used (23). Notably, when ovine derived cells were used in an *in vitro* model, virulence was restored in S strains (24). Thus far, these functional investigations of MAP have included a limited number of isolates, predominantly focused on differences between a few S and C strains (25–27). This is problematic, since S strains have been reported to be more heterogenic than C strains (13, 28).

Currently employed typing tools distinguish MAP isolates to varying degrees and numerous methods have been employed (29). IS*1311* restriction fragment length polymorphism (RFLP) is widely used for distinguishing between C and S strains of MAP (30). However, this technique is insufficient for distinguishing between the sheep MAP Type I and III sub-lineages. Strain typing of sheep MAP sub-lineages has been reported using the *gyrA* and *gyrB* genes. While these genes have a very low mutation rate and have previously been reported for typing of closely related mycobacteria (31), this test is not widely used for typing MAP or other mycobacteriaI species. Furthermore, few studies distinguish between sheep MAP types, thus presenting an information gap. Later studies investigating the genetic diversity of MAP utilised genotyping methods such as Mycobacterial Interspersed Repetitive Unit-Variable Number Tandem Repeat (MIRU-VNTR) and short sequence repeat typing (SSR) and other PCR assays (13, 14, 32, 33), which use multiple loci and are more useful for determining population structures. In one study, MIRU-VNTR typing was unable to distinguish between Type I and Type III sheep strains (13). However, tests such as MIRU-VNTR, SSR and PCR-based assays are able to distinguish MAP strains with higher efficiency when combined with each other or other techniques such as IS*900* RFLP (34–37). Despite these techniques being more advanced they still provide limited insight into the functional consequences of genetic diversity and have been shown at times to both underestimate and overestimate the diversity of MAP in some scenarios. In contrast, WGS enables high resolution genetic data to be obtained from bacterial isolates and enables more data to be obtained for each isolate than any other genotype test, leading to the resolution of relationships between lineages that has enabled a more complete overview of the population structure of MAP (9).

Studies on lineage-specific variants of MAP to date have focused on holistic differences between the S and C strains. Early literature utilised laborious subtractive hybridisation methods available at the time (38–41). This was followed by microarray hybridisation studies (42, 43). Today, WGS offers unique insights on comparative genomics. The first complete MAP whole genome sequence was on K10, a C strain isolate (44) and this is an invaluable resource for further comparative genomics work. In 2012, the draught sequence of S397, a Type III S strain, revealed differences between S and C strains at the whole genome level (45). Comparison of the K10 (type II) and S397 (type III) genome sequences revealed 10 large sequence polymorphisms in the type III isolate that contained >4 open reading frames, compared to the type II sequence (45). The presence of these polymorphisms from genomic data agrees with pan-genome microarray data (42, 43). Moreover, analysis of microarray suggests that there is significant variability between the sub-lineages of MAP S strains (43).

Understanding genetic diversity within a population of bacterial pathogens may provide insights into virulence, antibiotic susceptibility and other phenotypic traits important for the treatment and control of infectious diseases. Better characterisation of existing MAP strains will likely provides insights into mechanisms of host preference in S strains (25) and inform diagnostic test and vaccine development. Detected differences between the type I and type III sheep subtypes may reveal important evolutionary, epidemiological and virulence traits specific to each sub-lineage. In the present study, we compare type I and type III sheep MAP genomes from several global locations and host species using a pan-genome approach.

## Materials and Methods

### Isolate Collation

MAP genomes used in this study were from a variety of sources. Publicly available genomes were downloaded from the National Centre for Biotechnology Information (NCBI) GenBank and Sequence Read Archive (SRA) databases for *Mycobacterium avium* subspecies *paratuberculosis* on the 3rd of March 2020. Search philtres for genome, Illumina and DNA were used in the SRA. For isolates JQ5, JQ6, 88281, S397, JIII386 and Telford, raw reads were not available therefore assemblies were downloaded from GenBank. Additional New Zealand isolates AgS43 and AgS36 were originally sourced from sheep tissue or faecal samples from New Zealand Veterinary Pathology Limited (Palmerston North, New Zealand) and were regrown from the AgResearch Ltd. strain archive. Isolates 3410, 3443, 3413, 110b, 135b, 3324 and 3326 were sourced from the University of Sydney archive collection that were used in previous studies (22, 46). These isolates included four Australian isolates (3443, 135b, 110b, 3413 and 3410) and two Spanish isolates (3324 and 3326). Detailed information on isolates is available in Supplementary Material 1. Culture and extraction of isolates from the University of Sydney was done as described below.

### MAP Culture and DNA Extraction

Isolates were cultured as previously described (47) and pellets were washed and placed in 300 μL of Tris-EDTA (TE) (10 mM Tris, 1 mM EDTA, Ambion) buffer and stored at −80°C until further processing. MAP suspensions were thawed at room temperature prior to addition of 550 μL of TE buffer. Declumping was achieved by drawing the suspension through a 25-gauge needle seven times followed by vigorous vortexing. Suspensions were heat inactivated at 85°C for 30 min, then mechanically lysed in a 2 ml conical base screw capped tube containing 0.3 g of Zirconia/Silica beads (BioSpec Products Inc, Daintree Scientific) using a Tissue lyser II (Qiagen) at a frequency of 30 for 1 min 40 s, twice followed by centrifugation at 16,000 x *g* for 3 min and the supernatant was transferred to a new 1.5 ml tube. DNA extraction was performed based on the method of Choy et al. (21).

To disrupt the cell wall, 60 μL of 200 mg/mL Lysozyme (Sigma-Aldrich) was added and the samples were incubated for 2 h at 37°C with gentle mixing. To remove contaminating RNA, 20 μl 20 mg/ml RNAse (Sigma-Aldrich) was added and incubated for a further 3 h. To complete cell wall breakdown, 200 units of Mutanolysin (Sigma-Aldrich) was added and lysates were incubated for 12–16 h at 37°C with gentle mixing. Following this, 35 μL of Proteinase K solution (10 mg/mL) (Sigma-Aldrich) and 60 μL of 10% sodium dodecyl sulphate were added and the suspensions were incubated for 24 h at 37°C with gentle mixing. The Proteinase K was inactivated by heating at 70°C for 10 min and then 97.5 μL 5M NaCl and 82.5 μL CTAB/ NaCl (Bioline) pre-warmed to 65°C were added and the lysates incubated at 65°C with gentle mixing for 10 min. On completion, 700 μL of 25:24:1 phenol/ chloroform/ isoamyl alcohol (Sigma-Aldrich) was added and mixed vigorously for 30 s by pipetting. The upper aqueous phase was collected after centrifuging at 12,000 × g for 10 min. To remove excess phenol, an approximately equal volume of chloroform:isoamyl alcohol (24:1) (Sigma-Aldrich) was added and centrifuged at 12,000 x *g* for 10 min. The upper aqueous layer was collected and mixed well via inversion for 1 min with 1,000 μL of 2-Propanol (Sigma-Aldrich). DNA was pelleted by centrifuging for 15 min at 12,000 × g. The pellet was washed twice using 70% molecular-grade ethanol (Sigma-Aldrich) in nuclease-free water, cooled to −20°C, then centrifuged for 15 min at 12,000 × g. The supernatant was removed and the pellet was resuspended in 30 μL of 10 mM Tris buffer, pH 8.0 (Astral Scientific). Resolublization of DNA occurred at room temperature overnight with gentle mixing. On completion, samples were stored at −80°C. DNA quality was assessed using a NanoDrop^TM^ 2000 spectrophotometer (Thermo Fisher Scientific). Samples with A_260/280_ below 1.7 or A_230/280_ <1.2 were discarded and re-isolated. A Quant-iT™ PicoGreen™ dsDNA Assay Kit (Thermo Fisher Scientific) was used to measure DNA concentration.

New Zealand isolates AgS43 and AgS36 were re-cultured, extracted and sequenced as described by Gautam et al. (in preparation).

### Library Preparation and Whole Genome Sequencing

WGS of isolates 3410, 3443, 3413, 110b, 135b 3324 and 3326 was carried out at the NSW Mycobacterium Tuberculosis Reference Laboratory at the Centre of Infectious Diseases and Microbiology, Westmead Hospital on the Illumina sequencing platform. A Nextera XT library preparation kit (Illumina, Scoresby, Victoria, Australia) was used to generate paired indexed libraries of 150 base pairs in length as per the manufacturer's instruction. Sequencing was done using the Illumina NextSeq platform.

### Quality Control and Assembly

Fastq files were trimmed using Trimmomatic (version 0.36, RRID:SCR_011848) (48) with options set to -phred33, LEADING:3 TRAILING:3 SLIDINGWINDOW:4:20 MINLEN:36. Reads were assembled with SPAdes (version 3.12.0, RRID:SCR_000131) (49) using the default k-mer size testing options. To improve the assemblies, the Bayes-Hammer read correction, and careful option for post-assembly Burrows Wheeler Aligner mismatch correction (50) were also used. Seven isolates retrieved from GenBank were only available as assemblies (fasta files). Quality assessment of the assemblies was done with Quast (version 5.0.2, RRID:SCR_001228) (51). Assemblies which had a GC% of <69%, number of contigs >500 or a total length outside of 4.5–5.3 Mb were removed from the final analysis.

### Pan-genome Analysis

Genome annotation was undertaken with Prokka (version 1.13.3, RRID:SCR_014732) (52) with the minimum contig length set to 500 base pairs. GFF files from Prokka were used as an input for the Roary (version 3.12.0, RRID:SCR_018172) (53) pan-genome pipeline. Within this pipeline MAFFT (version 7.402) (54) was used to produce a nucleotide multifasta alignment of all core genes.

### Phylogenetic Analyses and Pruning

IQ-Tree (version 1.6.7, RRID:SCR_017254) (55) was used to generate trees from the core gene alignment output from Roary. Within IQ-Tree, ModelFinder (56) was used to identify the best-fitting model, which turned out to be the general time reversible model (GTR+F+R4) (57). Trees were visualised and annotated in iTOL (RRID:SCR_018174) (58). Any isolates that clustered with the S397 (accession AFIF01000001) or Telford (accession CP033688.1) reference genomes were retained and used in the downstream comparative analysis. Those which were phylogenetically distant to known sheep MAP genomes Telford (accession CP033688.1) (59) and S397 (accession AFIF01000001) or clustered with the K10 reference were discarded.

The K10 reference and any sequences that did not meet quality criteria outlined in the Quality control and assembly section were removed and Treemmer (60) was used to reduce redundancies within the type I dataset and bias of downstream analyses. Some manual selection of isolates was undertaken to maximise the geographical diversity of isolates within the dataset. The final dataset of 34 isolates contained 16 type I and 18 type III isolates. This enabled 95% of the original diversity to be retained. The K10 reference was retained in the final dataset as a root for the phylogenetic tree.

### Analysis of the gyrA/B Genes

The *gyrA* and *gyrB* genes from the Telford (accession CP033688.1) type I reference (59) and S397 (accession AFIF01000001) type III reference (45) genomes were used as a basis for *in silico* genotyping. BLAST analysis of the two versions of these two genes was used to confirm that the two major branches in the phylogeny were indeed Type I or Type III in the other analysed isolates. The online BLAST global align tool (available at https://blast.ncbi.nlm.nih.gov/Blast.cgi) from the National Centre for Biotechnology Information (NCBI) was used to compare the nucleotide and protein sequences of the genes between lineages.

### Querying the Pan-genome

**Figure 2:**
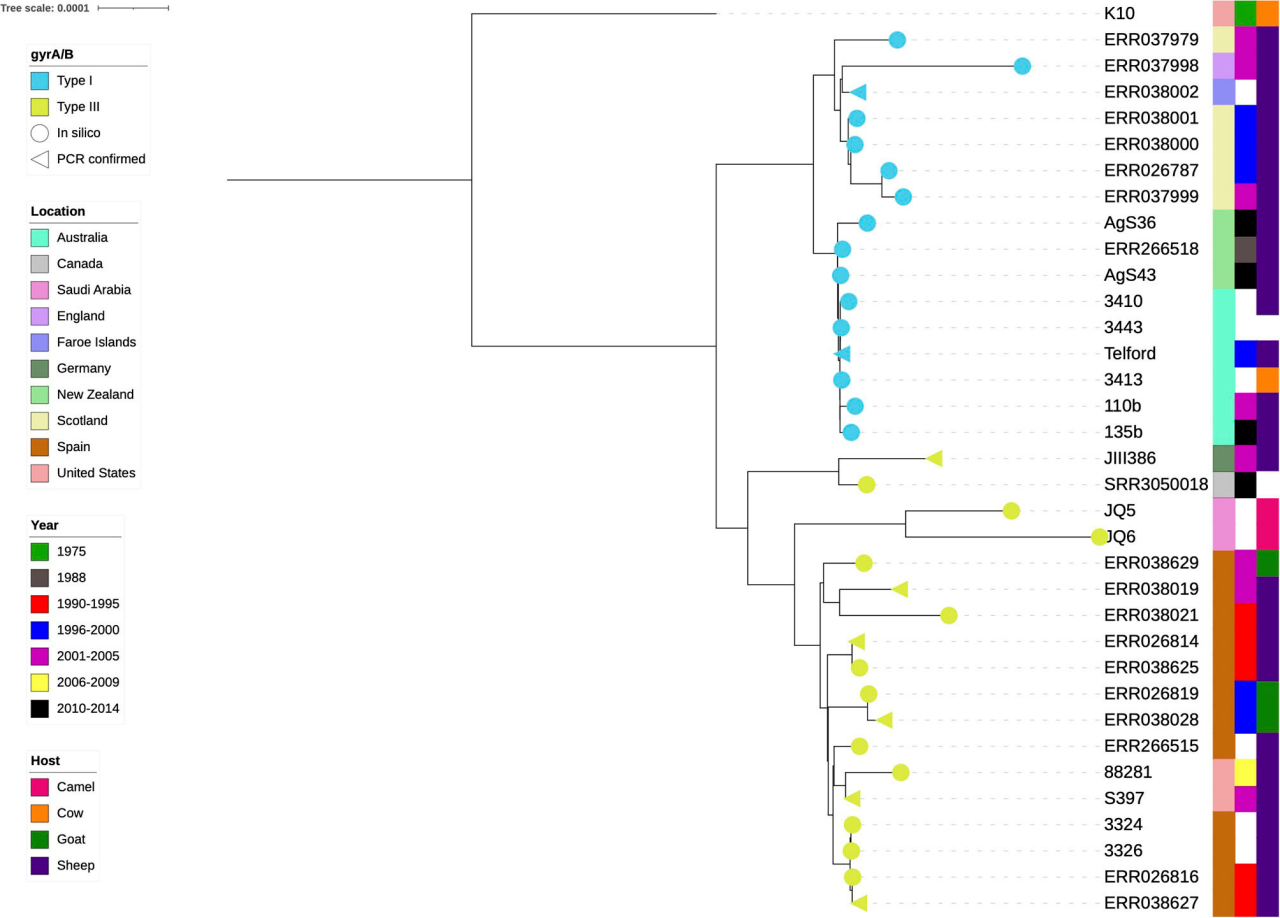
Phylogenetic tree of the final dataset of 34 sheep MAP isolates. The cattle K10 reference genome used in the tree as a root was excluded from all further analyses. Test confirmed types (*n* = 8) are annotated with a triangle, *in silico* results are represented by a circle, yellow is type III and blue is the type I. The colour strips from left to right are location, year of isolation and host species, respectively. White blocks indicate missing data.

The pan-genome analysis was repeated on the final dataset (Figure 2) to reduce the likelihood of noise due to misassembles or mis-annotations and prevent bias towards large clusters of highly similar genomes. Genes of interest were those that were present in one lineage and absent from the other or had consistent lineage-specific variations. To minimise the likelihood of a lineage-specific gene being an assembly or annotation artefact, candidate genes of interest had to be identified by Prokka annotation in all isolates from one lineage and completely absent from the other. The gene presence/absence output from Roary was used as an input for Scoary (61). This tool was used to identify genes of interest and demonstrate a gene's association with a respective lineage. A fasta file containing all lineage specific coding sequences (CDS) was uploaded to the EggNOG web-tool (available http://eggnog-mapper.embl.de/) (RRID:SCR_002456) to obtain functional categories for each gene. Contigs of draught genomes were reordered and aligned to the Telford reference genome (accession CP033688.1) with mauve **(**RRID:SCR_012852) (version 2.4.0) (62, 63) to view contig boundaries within isolates and confirm there were no contig boundaries interfering with the genes of interest.

### Blast

To obtain additional annotation data for hypothetical proteins potentially missed by automated annotation, a BLASTx of the nucleotide sequences of each gene of interest was undertaken using the NCBI online BLASTx tool (RRID:SCR_001653). The hit with the smallest e-value, a minimum of 99% identity and a minimum length of at least 99% of the query length was used. The nucleotide sequence of lineage-specific genes was obtained from the Roary pan-genome reference fasta output file. This file contains a representative nucleotide sequence for each protein annotated in the analysis. A nucleotide BLAST of each lineage-specific gene identified by Scoary to all sheep genomes in the study confirmed if the gene was present in the other lineage but had undergone mutations that led to an annotation failure by Prokka. The NCBI BLAST global align tool was used to compare lineage variants of protein sequences (available at https://blast.ncbi.nlm.nih.gov/Blast.cgi).

## Results

### Whole Genome Sequencing and Assembly

Approximately 400 MAP genomes were available from the SRA and an additional 50 were available from GenBank. These genomes were screened and assemblies that did not reach quality thresholds described in the methods quality assessment section and those that clustered more closely with K10 than S Type references were removed. Treemmer culled an additional 40 S strain isolates that represented redundancies in the dataset. The final dataset included 16 Type I and 18 Type III isolates from 10 different geographical sources and five different hosts. The average GC% content across both lineages was 69.23% (Table 1). The average genome length was 4,819,192 bp and 4,794,996 bp for Type I and Type III isolates, respectively (Table 1). Overall, the average quality of the draught assemblies was similar with the number of contigs and N50 for Type I at 268.9 and 45,862 bp and 247.9 and 113,636.8 bp for Type III. Detailed information on isolate-specific assembly statistics from QUAST and strain metadata can be found in Supplementary Material 1. A phylogenetic tree including public sequences culled by Treemmer to optimise diversity and those that did not meet assembly quality thresholds is available in Supplementary Material 2.

**Table 1:** Average basic assembly metrics and statistics for the isolates in this study and comparison of the sub-lineages.

	K10*	**All sheep**	**Type I**	**Type III**
No. genomes	1	34	16	18
GC%	69.3	69.23	69.24	69.22
Genome length (bp)	4,829,781	4,807,094	4,819,192.25	4,794,996
No. Contigs	1	258.38	268.87	247.89
N50**	4,829,781	79,749.39	45,862	113,636

Metrics were calculated on the final dataset after pruning with Treemmer and removal of assemblies which did not meet the quality standards of GC% > 69%, number of contigs <500 or a total length within 4.5–5.3 Mb. All figures are to two decimal places.

*Only one genome in this category, the K10 C strain reference, therefore these figures are not averages.

*N50 is the length (in base pairs) of the shortest contig at 50% of the total genome length.

### Analysis of gyrA/B Genes

Eight isolates had previously undergone lineage typing using the *gyrA/B* genes using PCR and sequencing (9, 45, 59, 64, 65). The *gyrA*/*B* BLAST results of the present study were in agreement with these results. Only one *gyrA/B* type was present per lineage within the phylogeny (yellow and blue branch symbols, Figure 2).

A BLASTp global alignment between the *gyrA* translated proteins from the Type I (Telford, CP033688.1) and Type III (from S397, AFIF01000001) reference genomes revealed a single mismatch where the Type I reference contained a glutamic acid instead of a lysine at position 290. Alignment of the *gyrB* protein sequences revealed a glutamic acid instead of a lysine at position 594 in the Type I reference. This confirms one of the SNPs found in each nucleotide sequence is non-synonymous. No differences in gene or protein length were seen between the Type I and Type III.

Most of the type I isolates had a *gyrA* and *gyrB* nucleotide sequence that was identical to the type I reference genome. The one exception was the New Zealand isolate AgS36 which had a single, synonymous SNP in both the *gyrA* and *gyrB* gene. All Type I *gyrA* and *gyrB* protein sequences were identical.

Type III isolates contained two (*n* = 16 isolates) or three (*n* = 2 isolates) SNP differences compared to the type I *gyrA* gene. The type III nucleotide sequence of the *gyrA* gene was consistently different by two SNPs with all type I isolates. Within the Type III isolates, the S397 *gyrA* gene was 100% identical to 16 isolates and had a single SNP difference compared to two of the type III isolates. The two type III isolates which contained an additional SNP in the *gyrA* gene were JIII386 and SRR3050018.

Protein sequence BLASTs revealed a single mismatch between the Type I *gyrA* reference sequence and 16 Type III isolates. Type III isolates JIII386 and SRR3050018 had two mismatches compared to the type I *gyrA* which also had a mismatch to the type III *gyrA* protein sequence indicating a non-synonymous mutation. Protein sequences from these two isolates were identical. Alignment of the S397 *gyrA* protein to that of isolate JIII386 demonstrated a single mismatch of an arginine to a glycine at amino acid number 558. A schematic comparison of the Type I and III reference *gyrA* proteins to that of JIII386 is available in Supplementary Material 3.

The nucleotide sequence of the *gyrB* gene from the Type I reference genome differed by 2 or 3 SNPs compared to all Type III isolates. The nucleotide sequence of the type III *gyrB* gene from the Type III reference genome was two SNPs different to all type I isolates, 100% identical to 16 of the type III isolates and differed by one SNP in isolates JQ5 and JQ6. The protein sequence of all Type III isolates was identical, indicating that the SNP in isolate JQ5 and JQ6 was synonymous.

Isolates JIII386, SRR3050018, JQ5 and JQ6 were within the Type III cluster (Figure 2), their *gyr* genes more closely resembled the Type III lineage and previous typing of JIII386 (65), JQ5 and JQ6 (64) indicated they were Type III, thus in this investigation they were considered Type III.

### Phylogeny

A clear distinction between the Type I and Type III isolates became obvious when the K10 reference was used as a tree root (Figure 2). The type I isolates were of Australian, New Zealand and European origin, with a distinct and very closely related Australia and New Zealand clade present. The type III isolates appeared more diverse, both genetically as indicated by their branch lengths in the phylogenetic tree, and geographically.

### Pan-genome Analysis

**Figure 3:**
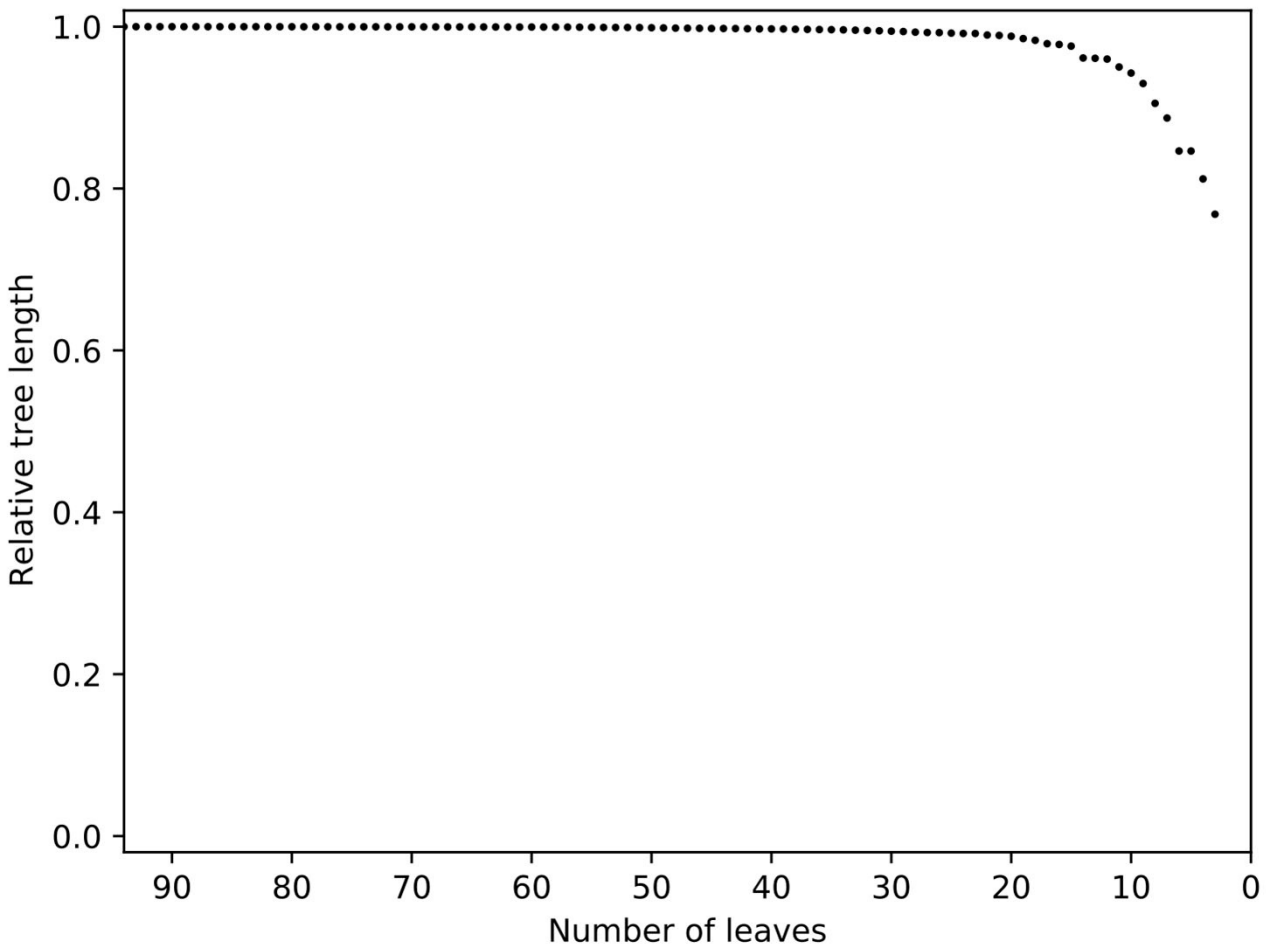
Relative tree length plot of the dataset prior to trimming with Treemmer. Number of leaves is equal to the number of genomes present in a phylogenic tree. Note the steep curve indicating that a small number of genomes represents a large amount of the diversity in this dataset. Additional genomes in this study would not have increased the genetic diversity within the dataset, thus redundant genomes were removed. K10 was not included in this process since it is an outlier in the tree.

The increase in pan-genome size with the addition of new isolates or strains can be used to predict the discovery rate of new genes within a species (66). The initial tree demonstrated that several isolates were very distant (data not shown) and contrary to their labelling were unlikely to be MAP. Others clustered with the K10 reference genome, indicating they were likely to be C strains. After removal of C strains, distant isolates and those that did not meet the quality thresholds outlined earlier, the resulting phylogeny had one very large, flat clade of Australian isolates indicating low diversity and a potentially skewed dataset to the type I isolates (data not shown). A relative tree length plot from Treemmer (Figure 3) revealed low diversity of MAP isolates and many redundant sequences. Overall within the final dataset, the core genome (genes in 100% of isolates) contained 3,239 genes, soft core genome (95–99%) of 423 genes, accessory genome 1,408 and cloud genome (<15% of isolates) had 2,582 genes (Table 2).

**Table 2:** Number of genes present in each category of genes in the pan-genome categorised by Roary.

**Category**	All^*^
Core (99–100%)	3,239
Soft core (95–99%)	423
Accessory (15–95%)	1,408
Cloud (<15%)	2,582
Total	7,652
No. genomes	34
No. genes of interest	13

Genes are categorised by Roary into core, soft core, accessory and cloud genes.

* *These summary statistics exclude the K10 C strain reference genome*.

### Lineage-specific Genes and Variations

Scoary identified 13 candidate lineage-specific genes, with 9 in the Type I and four in the Type III isolates. All annotated genes of interest had a sensitivity and specificity of 100% and a Bonferroni corrected *P*-value of 4.54 × 10^−10^. As described in the methods, genes of interest were those that were present in one lineage and absent from the other or had consistent lineage-specific variations. BLAST analysis demonstrated that the lineage-specific genes called by Scoary were present in all isolates but contained consistent SNP variations and this had resulted in different annotations for these genes (Table 3).

**Table 3:** Lineage-specific genes and variation of type I and type III isolates.

**Gene**	**Type**	**Annotation**	Variations^*^
Group 4585	I	Putative nuclear transport factor 2 family protein	5 variable mismatches in the Type III protein
Group 4593	I	MMPL family transporter	No significant protein hit in Type III isolates
*cinA*1	I	1,8-cineole 2-endo-monooxygenase	Type III protein has 8–17 mismatches, length is identical between lineages
*mhpA*2	I	3-(3-hydroxy-phenyl)propionate/3-hydroxycinnamic acid hydroxylase	Type I isolate protein sequences are 62 amino acids longer and Type III isolates have a single mismatch
Group 4493	I	Hemolysin III family protein	Type III protein is 54 amino acids longer and contains 9 mismatches to the Type I version
Group 4363	III		
Group 4592	I	MMPL family protein	No significant protein hit in Type III isolates
Group 1815	I	Hypothetical protein	1-2 mismatches in Type III protein and Type III is 22 amino acids shorter
Group 4617	I	*TetR/AcrR* family transcriptional regulator	11 amino acid mismatches and the Type I protein is three amino acids shorter
Group 4778	III		
Group 4500	I	Nitroreducatase family protein	Type I protein is 185 amino acids long and Type III is 171. Contains 11 mismatches
Group 4772	III		
Group 4781	III	Hypothetical protein	Type I isolates 37–164 amino acids long with 24–105 mismatches. Type III are all 299 amino acids long with a single mismatch present in four isolates

Where Prokka annotated the gene as a hypothetical protein but BLASTx was able to provide a putative annotation, the BLASTx annotation was used. Variations are from BLASTp results.

* *bp = base pairs*.

**Figure 4:**
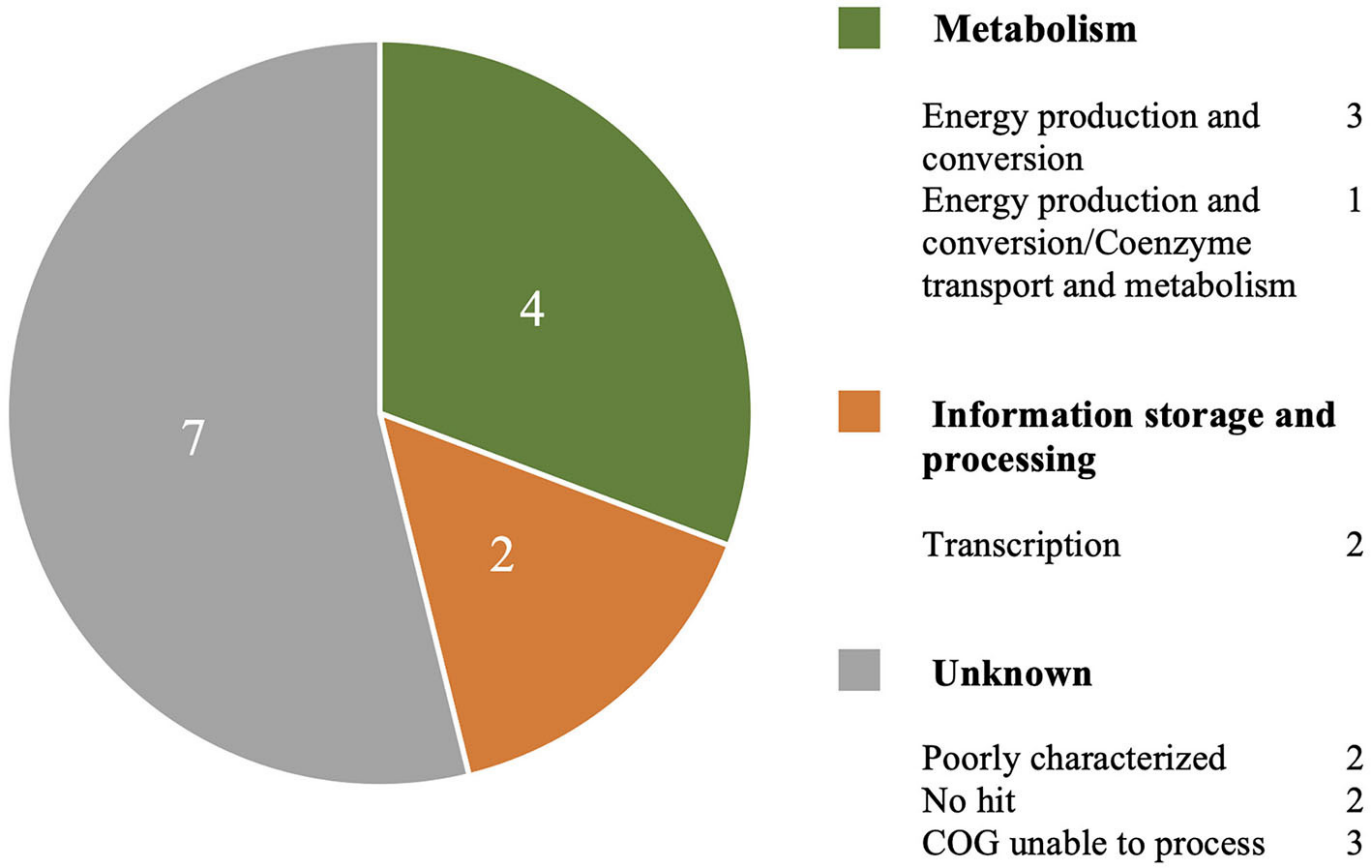
A breakdown of COG annotations for genes of interest from Type I and Type III S strain lineages.

The MAUVE alignment revealed no contig boundaries present in the genes of interest. The Type I genes of interest included *cinA1, mphA2* and seven hypothetical proteins. The Type III genes included four hypothetical proteins. BLASTx analysis of nucleotide sequences retrieved from the Roary pan-genome reference fasta file revealed additional annotations (Table 3). BLASTx results are available in Supplementary Material 4. Six genes were identified that are involved in metabolism, information processing and storage. Seven of the genes were uncharacterised and COG categories are unavailable (Figure 4).

Most genes called in the Type I lineage were present in type III, but due to inconsistent differences within the type III isolates, they were not recognised as lineage specific for type III (Table 3). This was found in the putative nuclear transport factor 2 family protein (group 4585), hypothetical protein 1815 and *cinA1*. Differences in the *mhpA2* protein were consistently different between Type I and III isolates. However, the very large difference in length between Type I and Type III BLAST result indicates this may be an inconsequential alignment in the Type III lineage. Type III specific hypothetical protein 4781 had no significant hits in 10 Type I isolates and hits with 82–105 mismatches in the remaining isolates. Some SNPs were present in group 4781 (hypothetical protein) within the Type III lineage, resulting in a single mismatch that did not affect the overall length of the protein (Table 3).

Some genes had similar BLASTx results and appeared to be variants of the same gene in each lineage. The Type I lineage-specific haemolysin family protein (group 4493) is 203 amino acids in length and 79% identical to the Type III haemolysin. The Type III haemolysin family protein (group 4636) is 257 amino acids in length and contains 9 mismatches compared to the Type I version. The TetR/AcrR family transcriptional regulator is 80% identical between lineages and differs by 11 mismatches and three amino acids in length. The nitroreducatase family proteins are 82% identical, differ by 11 amino acid mismatches and the Type III protein is 14 amino acids shorter. Two type I hypothetical proteins, group 4593 and group 4592 had no significant protein hit in type III isolates. The haemolysin III family protein found in both lineages was also discovered in a previous study (MAP2704) (43). BLASTx of MAP2704 produced similar results to that of the Type III variant (group4363). A summary of lineage-specific variants that match previously identified variable loci and a copy of Table 3 that summaries the differences between Type I and Type III genes of interest that also includes differences found in the K10 reference strain is available in Supplementary Material 5.

## Discussion

Most studies on Johne's disease and MAP epidemiology do not distinguish between S sub-types and thus the relative abundance and virulence of each is not widely known. In this investigation, we address the epidemiology of each of these sub-types with the view that conserved differences detected between lineages could be used for future studies into comparative virulence and larger epidemiological investigations. We also attempted to reveal genomic differences using a pan-genome approach.

The current test used to distinguish between Type I and Type III lineages of sheep MAP uses a PCR-based assay on the *gyrA/B* genes. Previously, these genes were demonstrated to have a low mutation rate and be ideal for the typing of slow growing mycobacteria (31). The presence of variable SNPs in these genes, which were identified in our study, indicate that they are less conserved than suggested by Kasai and colleagues. This variability may cause problems if SNPs occur in the primer binding sites of the existing assay (67). Thus, additional markers identified by the present study may be useful.

Several attempts to understand the genetic diversity of MAP have been undertaken (9, 14, 33, 36, 68). Of those using WGS as a tool (9, 68), most isolates have been C strains (Type II lineages). These investigations have incorporated several human isolates, which have all clustered with the C strains of MAP (9, 69). One possible reason for this is that C strains have a broader host preference, while S strains of MAP are generally more host-specific, though still capable of cross-species transmission given the appropriate host-pathogen interactions and level of infection pressure (14, 20). Due to the relatively small number of S strains included in the published studies to date, minimal conclusions could be drawn for S strains of MAP. A major limitation of earlier studies is the use of culture media that did not support the growth of S strains of MAP (47). This may have led to a bias for inclusion of mainly C strains of MAP in the early studies and in contemporary studies which have used inappropriate culture media. Routine inclusion of S strain isolates in typing studies and improved ability to recognise the two sub-lineages may assist with tracing an S strain isolate in epidemiological investigations including human infections. Furthermore, historical (20) and contemporary reports of cross-species transmission of S strains in co-grazing properties (14) leading to economic losses and between wildlife reservoirs (16, 70) means that these strains have genuine relevance.

Overall, the Type III lineage isolates analysed here appeared to be more genetically heterogeneous than the Type I lineage but were derived from a broader array of geographical sources. Isolates of the Type III sub-lineage represent multiple continents including America, Europe and Asia in their locations. The Type III lineage had a larger number of genes in the pan-genome, displayed longer branches on the tree, and only four lineage-specific genes were identified. In contrast, Type I isolates were less diverse and had a larger number of genes characterised as being in the core genome. These findings are supported by previous work, which found Type I isolates to be more homogenous (71). Similarly, the finding that Type III isolates are more heterogeneous than Type I isolates is also in agreement with earlier findings which utilised PFGE and *IS*900-RFLP typing techniques on a panel of isolates from a variety of countries (13, 28).

There appears to be association of lineage Type with different regions of Europe, with all Spanish and German isolates belonging to the type III lineage and all European type I isolates from Scotland, England and the Faroe Islands. One finding that was not supported by literature was that Type III is the predominant Type throughout the United States of America. Previous work using SSR and PFGE identified Type I isolates to be the predominant type throughout the United States (71). This discrepancy is potentially due to only two sheep MAP whole genome sequences being available from this location. Overall, these epidemiological findings must be interpreted with caution due to the small number of isolates used in the present study. Other biases include a lack of culturability of certain types of MAP, and both the sampling effort in particular countries and enthusiasm of people who have gathered and curated culture collections to facilitate such studies. These factors may introduce sampling bias to the apparent epidemiology of sheep MAP Types illustrated here.

Within Oceania, this study found low diversity in sheep MAP. This may reflect a small sample size of the present study or the slow rate that MAP accumulates genetic variation (9). A similar conclusion for sheep MAP isolates was found in an earlier study that utilised *IS*900 RFLP and *IS*1311 polymorphism analyses of an Australia-wide panel of isolates. Only a single RFLP type (S1) was found in sheep strains (12). A recent epidemiological study of S strains of MAP in New Zealand using VNTR/SSR (14) and WGS (Gautam et al., in preparation) demonstrated low sequence diversity across the country. Akin to the present study, New Zealand isolates were solely of the Type I lineage and Australian and New Zealand isolates appeared to be closely related. Of relevance is that our methods differed from those of the New Zealand group, which mapped all isolates to the Telford reference genome, whereas the present study utilised *de novo* assembly. The similar grouping of sheep isolates from Australia and New Zealand in comparison to those from other countries helps validate both approaches when exploring MAP diversity. The tight clustering of Australian and New Zealand sheep isolates may indicate recent transmission between these countries and reflect geographical isolation from the rest of the world.

Thirteen genes were annotated as lineage-specific in this investigation, using the program Scoary. BLAST results demonstrated that each of these are not fully lineage-specific genes, but that each gene contained a lineage-specific mutation. Discrepancies between Roary and BLAST are potentially due to small variants within genes, such as insertions or deletions. These mutations may have moved the reading frame of the nucleotides, leading to alternate predicated protein sequences such that annotation software no longer recognised these proteins as being derived from the same gene. Eleven of the 13 genes of interest were annotated as hypothetical proteins, and in these cases, BLASTx was used to determine if they had functions previously identified in other, related species. BLASTx also served to find overlaps between lineage-specific genes from each group, where annotations from Prokka were ambiguous. Previous microarray data demonstrated several variable loci between Type I and Type III isolates (43). Many of these findings were supported by the present study including one locus (MAP2704) that encodes a haemolysin III family protein that was found in both studies (group4363 in Type III and group4493 in Type I). BLASTx of the MAP2704 nucleotide sequence retrieved an identical result as the Type III specific gene group4363 (Supplementary Material 5). MAP2325 was thought to be absent from Type I isolates (42) but was later found in Type I isolates from countries other than Australia (43). This gene was absent from all Australian Type I isolates tested but present in all other isolates with a single SNP present in some Type III isolates.

Type I isolates contained more conserved unique variants than Type III isolates, potentially due to their lower diversity. Proteins derived from most of these genes were found in the Type III isolates, but in the other lineage the sequences contained SNPs and polymorphisms which were inconsistent between isolates, whereas all the Type I sequences were identical. Genes in Type I isolates that were variable in Type III included a lineage-specific putative nuclear transport factor 2 family protein, mycobacterial membrane protein large (MMPL) family transporter, MMPL family protein, hydroxycinnamic acid hydroxylase (*mhpA*2) and a monooxygenase (*cinA*1). The MMPL genes are a subgroup of resistance-nodulation-division transporters involved in trans-envelop and trans-membrane export of immunomodulatory lipid components in mycobacteria (72). Prior investigations discovered variation in these genes in S strains of MAP (38). Both MMPL proteins from the Type I isolates had no significant hits in the Type III isolates and no hits to any MAP genomes in the NCBI BLASTp database. The role of these proteins in lipid export would require further investigation in both lineages. The *mphA* genes have not been extensively studied in mycobacteria but are involved in redox reactions (73). Similarly, the monooxygenase is a member of a superfamily of haemoprotein enzymes responsible for oxidative metabolism of fatty acids and acyl homoserine lactones (74). These enzymes are thought to give bacteria a competitive advantage since they can block signalling of other bacteria (75). Differences in these genes between lineages may offer a regional selective advantage against conditions encountered by each lineage. Alternatively, the higher similarity of Type I isolate may indicate clonal expansion of a more virulent isolate that diverged from the common ancestor of Type I and Type III isolates in the past.

Both lineages had consistent variations in genes involved in metabolism and transcription including a haemolysin III family protein, a nitroreducatase family protein and a *TetR/AcrR* family transcriptional regulator. These genes have all been previously linked with virulence. Haemolysin III family proteins are surface associated and may be involved in the acquisition of nutrients and drug resistance in *M. tuberculosis* (76). Nitroreductase enzymes convert nitro-containing compounds to their corresponding amine and are associated with the stress response in mycobacteria (77). The *TetR/AcrR* family transcriptional regulator is a gene involved with a paired mechanism responsible for chemical signalling and bacterial homeostasis (78). Further *in vitro* work would be required to investigate if these differences have a functional impact on MAP lineages.

An unexpected finding in the present study was the variability of the *gyrA* and *gyrB* genes within sub-lineages. These genes have been described to have type-specific mutations between lineages (67). Non-synonymous SNPs were found within the type III isolates in the *gyrA* gene and synonymous SNPs found in the *gyrB* gene. These mutations may represent regional variants. Sequencing of PCR amplicons may have diagnostic value, but further work is needed to characterise the extent of these mutations in various regions. A lineage-specific *gyr* variant was not found by the pan-genome analysis, since the resulting proteins were of the same length between the lineages and thus annotation software successfully identified the proteins as the same. These genes have been the subject of multiple studies in *M. tuberculosis*, in which SNPs have been found to confer antimicrobial resistance (79, 80). Antimicrobial resistance is not typically a concern in MAP since the use of antibiotics to treat Johne's disease in ruminants is uncommon. However, keeping track of potential resistance markers may have human clinical relevance. The presence of within-lineage SNPs in these genes indicates that their use as lineage-specific markers may not be ideal and a new diagnostic test may be required for rapid lineage identification.

Initially, *de novo* assembly was chosen over mapping to a reference genome, since a wider variety of isolates were being used and a single reference genome was unlikely to be appropriate for all isolates. At the time of this study, only a single reference genome was complete, the Australian Telford Type I (CP033688.1), all other available sheep strains were only available as draught assemblies. Using a reference genome on isolates that are diverse could have led to unmapped regions being missed by the analysis. To reduce the possibility that our genes of interest were assembly or annotation artefacts, genes annotated by Prokka had to be identified by Scoary as lineage-specific. These candidate genes of interest were further investigated by BLAST to confirm that within a lineage they were identical and that between lineages, there were consistent differences specific to their respective lineages.

Due to the possibility of assembly errors, poor and/or incomplete coverage, more lineage specific genes and variations may be present but were not identified by the present study. Repetitive regions such as the PPE/PE genes are difficult to assemble since the assembly software cannot be certain where raw sequencing reads belong if two regions are highly similar or repetitive. These genes have been the subject of numerous studies and can represent important virulence traits and genomic variation (81–86). Assembly errors in regions such as these can result in a loss of important information on isolate diversity. Efforts were made to include only high-quality genomic data in the study. Nevertheless, more lineage variations may have been missed due to their presence on a contig boundary or poor genome coverage in particular regions such as the PPE/PE genes of some isolates. This was partly seen in several genes that were identified using Scoary as lineage-specific in type I isolates but not type III, due to slight differences within the Type III group. Future investigations may require long read sequencing such as Nanopore long read sequencing so that reads span regions that are difficult to assemble, such that repetitive regions can be accurately assembled (87). This would enable the construction of complete sheep genomes so that more detailed comparisons can be made, a method which was recently employed to compare genomes across all of *M. avium* (88). Having a number of complete or closed sheep MAP genomes has the potential to reveal important differences in that we were unable to be identify.

## Conclusion

Within sub-lineages of S strain MAP, there appear to be distinct regional clusters, such as the Australian-New Zealand group within the Type I lineage. The Type I lineage exhibits low genetic diversity compared to the Type III lineage. Within lineages, there is evidence for lineage-specific variants of genes associated with virulence in mycobacteria. By revealing lineage-specific markers for S strains of MAP, there is potential for improving diagnostics so that rapid identification of Type I and III strains can occur. These *in silico* findings require further testing *in vitro* prior to the development of a lineage-specific diagnostic test. This knowledge could reveal insights into the epidemiology and spread of these lineages without the need for full genome sequencing in future studies. Further *in vitro* work may assist in identifying the functional differences of these genes and reveal how lineage differences relate to virulence and host adaptation.

## Data Availability Statement

The datasets generated for this study can be found in online repositories. The names of the repository/repositories and accession number(s) can be found below: NCBI Sequence Read Archive, SRR13214442 - SRR13214448.

## Author Contributions

RM was responsible for the data collection, bioinformatics analysis and writing of the manuscript. VT and KP assisted with the study design and editing of the manuscript. VT also assisted with the bioinformatics analyses. RW assisted in the collection, curation and description of some of the study isolates and editing of the manuscript. MP-C, MG, PB, and CH assisted with obtaining isolates and metadata from New Zealand and editing of the manuscript. All authors contributed to the article and approved the submitted version.

## Conflict of Interest

The authors declare that the research was conducted in the absence of any commercial or financial relationships that could be construed as a potential conflict of interest.

## Supplementary Material

Supplementary Material 1 Isolate metadata list and QUAST results Excel spreadsheet.

Supplementary Material 2 Phylogenetic tree including public sequences culled by Treemmer and those that did not meet assembly quality thresholds.

Supplementary Material 3 Type I and III reference and JIII386 *gyrA* protein alignment schematic.

Supplementary Material 4 BLASTx results of lineage-specific genes.

Supplementary Material 5 Previously identified variable loci from Castellanos et al. (43) in sheep MAP strains.
