# Supplementary material for: Comparative Genomics of Mycobacterium avium Subspecies Paratuberculosis Sheep Strains
Source: Front Vet Sci. 2021 Feb 15;8:637637. doi: 10.3389/fvets.2021.637637 (PMC7917049; doi:10.3389/fvets.2021.637637)
Supplement: Supplementary Material 2 — Phylogenetic tree including public sequences culled by Treemmer and those that did not meet assembly quality thresholds. [file Data_Sheet_2.pdf]

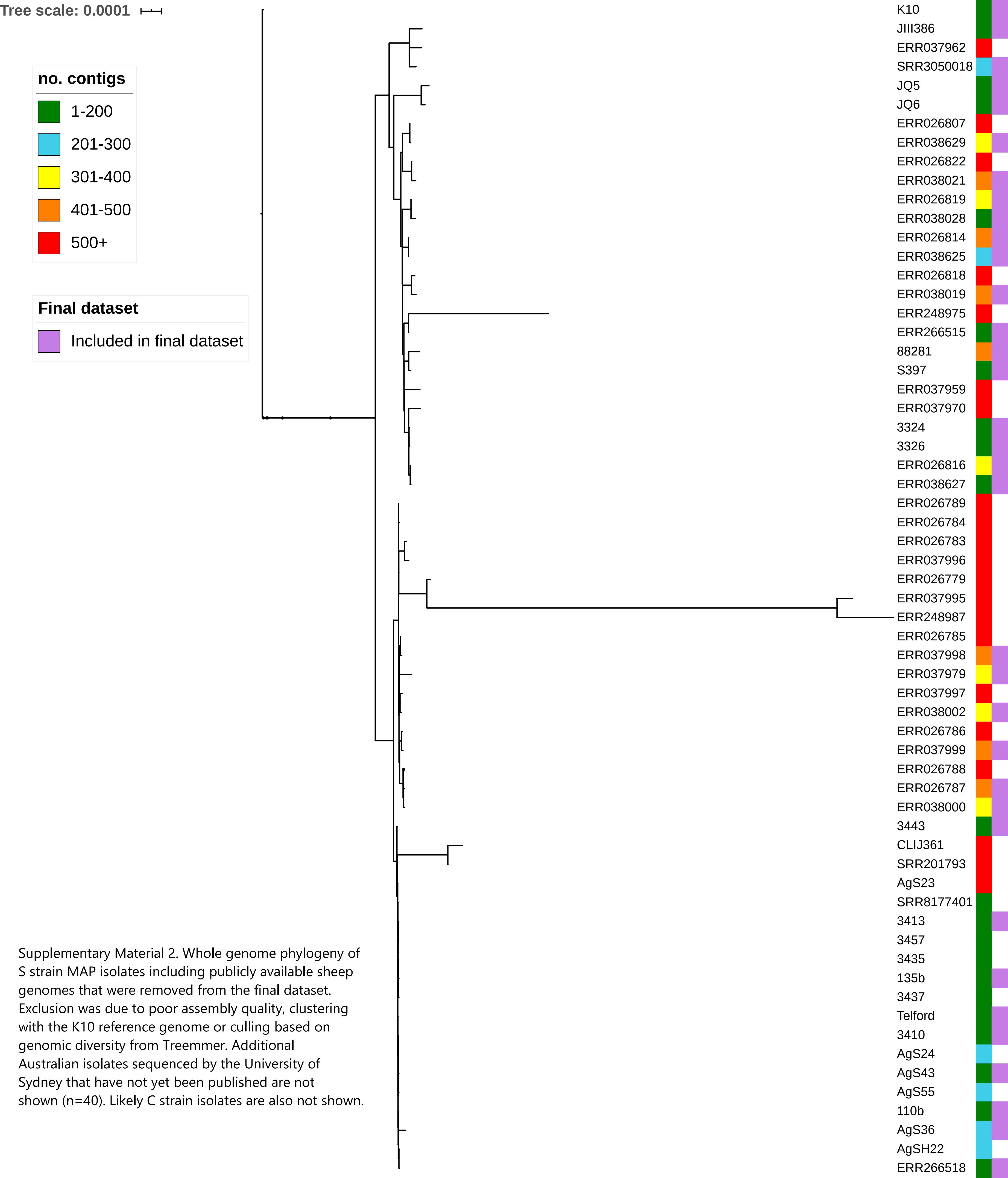

Supplementary Material 2. Whole genome phylogeny of S strain MAP isolates including publicly available sheep genomes that were removed from the final dataset. Exclusion was due to poor assembly quality, clustering with the K10 reference genome or culling based on genomic diversity from Treemmer. Additional Australian isolates sequenced by the University of Sydney that have not yet been published are not shown (n=40). Likely C strain isolates are also not shown.
