# Supplementary material for: Comparative Genomics of Mycobacterium avium Subspecies Paratuberculosis Sheep Strains
Source: Front Vet Sci. 2021 Feb 15;8:637637. doi: 10.3389/fvets.2021.637637 (PMC7917049; doi:10.3389/fvets.2021.637637)
Supplement: Supplementary Material 3 — Type I and III reference and JIII386 gyrA protein alignment schematic. [file Data_Sheet_3.docx]

Supplementary material. 3

Protein alignment of the *gyrA* protein sequence from the Type I reference genome (Telford, CP033688.1)(top), Type III reference genome (middle) and Type III isolate JIII386 (bottom). The lysine to glutamic acid mutation at amino acid 290, previously described by Castellanos et al. (2007) was present in all Type III isolates in the present study. The atypical arginine to glycine change at amino acid 558 was only seen in two (JIII386 and SRR3050018) of the 18 Type III isolates in the study. Figure not to scale.

Telford (CP033688.1)

|  |  |  |  |  | K |  |  |  |  |  |  |  |  |  |  |  |  |  |  |  |  |  | R |  |  |  |  |  |  |  |  |  |
| --- | --- | --- | --- | --- | --- | --- | --- | --- | --- | --- | --- | --- | --- | --- | --- | --- | --- | --- | --- | --- | --- | --- | --- | --- | --- | --- | --- | --- | --- | --- | --- | --- |

S397 (accession)

|  |  |  |  |  | E |  |  |  |  |  |  |  |  |  |  |  |  |  |  |  |  |  | R |  |  |  |  |  |  |  |  |  |
| --- | --- | --- | --- | --- | --- | --- | --- | --- | --- | --- | --- | --- | --- | --- | --- | --- | --- | --- | --- | --- | --- | --- | --- | --- | --- | --- | --- | --- | --- | --- | --- | --- |

JIII386 (accession)

|  |  |  |  |  | E |  |  |  |  |  |  |  |  |  |  |  |  |  |  |  |  |  | G |  |  |  |  |  |  |  |  |  |
| --- | --- | --- | --- | --- | --- | --- | --- | --- | --- | --- | --- | --- | --- | --- | --- | --- | --- | --- | --- | --- | --- | --- | --- | --- | --- | --- | --- | --- | --- | --- | --- | --- |

Legend

|  | Identical amino acid |
| --- | --- |
|  | Previously described Type variant |
|  | Novel variant |

Supplementary material. 3 BLASTp full alignment of the *gyrA* protein sequence from the Type I reference genome (Telford, CP033688.1)(Query) and *gyrA* protein sequence from the Type III isolate JIII386 (subject). Isolate JIII386 contains the atypical arginine to glycine change at amino acid 558 (yellow highlight). This mutation was only seen in two of the 18 Type III isolates in the study. In addition, this Type III isolate contains the lysine to glutamic acid mutation at amino acid 290, previously described by Castellanos et al. (2007)(green highlight). This mutation was present in all Type III isolates in the present study.

Query 1 MTDTTLPPGGDAADRVEPVDIQQEMQRSYIDYAMSVIVGRALPEVRDGLKPVHRRVLYAM 60

MTDTTLPPGGDAADRVEPVDIQQEMQRSYIDYAMSVIVGRALPEVRDGLKPVHRRVLYAM

Sbjct 1 MTDTTLPPGGDAADRVEPVDIQQEMQRSYIDYAMSVIVGRALPEVRDGLKPVHRRVLYAM 60

Query 61 YDSGFRPDRSHAKSARSVAETMGNYHPHGDASIYDTLVRMAQPWSLRYPLVDGQGNFGSP 120

YDSGFRPDRSHAKSARSVAETMGNYHPHGDASIYDTLVRMAQPWSLRYPLVDGQGNFGSP

Sbjct 61 YDSGFRPDRSHAKSARSVAETMGNYHPHGDASIYDTLVRMAQPWSLRYPLVDGQGNFGSP 120

Query 121 GNDPPAAMRYTEARLTPLAMEMLREIDEETVDFIPNYDGRVQEPTVLPSRFPNLLANGSG 180

GNDPPAAMRYTEARLTPLAMEMLREIDEETVDFIPNYDGRVQEPTVLPSRFPNLLANGSG

Sbjct 121 GNDPPAAMRYTEARLTPLAMEMLREIDEETVDFIPNYDGRVQEPTVLPSRFPNLLANGSG 180

Query 181 GIAVGMATNIPPHNLGELAEAVFWALDNYEADEEATLAAVMERVKGPDFPTSGLIVGTQG 240

GIAVGMATNIPPHNLGELAEAVFWALDNYEADEEATLAAVMERVKGPDFPTSGLIVGTQG

Sbjct 181 GIAVGMATNIPPHNLGELAEAVFWALDNYEADEEATLAAVMERVKGPDFPTSGLIVGTQG 240

Query 241 IADAYKTGRGSIRMRGVVEVEEDSRGRTSLVITELPYQVNHDNFITSIAKQVRDGKLAGI 300

IADAYKTGRGSIRMRGVVEVEEDSRGRTSLVITELPYQVNHDNFITSIA+QVRDGKLAGI

Sbjct 241 IADAYKTGRGSIRMRGVVEVEEDSRGRTSLVITELPYQVNHDNFITSIAEQVRDGKLAGI 300

Query 301 SNIEDQSSDRVGLRIVIELKRDAVAKVVLNNLYKHTQLQTSFGANMLAIVDGVPRTLRLD 360

SNIEDQSSDRVGLRIVIELKRDAVAKVVLNNLYKHTQLQTSFGANMLAIVDGVPRTLRLD

Sbjct 301 SNIEDQSSDRVGLRIVIELKRDAVAKVVLNNLYKHTQLQTSFGANMLAIVDGVPRTLRLD 360

Query 361 QLIRHYVDHQLDVIVRRTTYRLRKANERAHILRGLVKALDALDEVIALIRASETVDIARQ 420

QLIRHYVDHQLDVIVRRTTYRLRKANERAHILRGLVKALDALDEVIALIRASETVDIARQ

Sbjct 361 QLIRHYVDHQLDVIVRRTTYRLRKANERAHILRGLVKALDALDEVIALIRASETVDIARQ 420

Query 421 GLIELLDIDEIQAQAILDMQLRRLAALERQRIIDDLAKIEAEIADLEDILAKPERQRGIV 480

GLIELLDIDEIQAQAILDMQLRRLAALERQRIIDDLAKIEAEIADLEDILAKPERQRGIV

Sbjct 421 GLIELLDIDEIQAQAILDMQLRRLAALERQRIIDDLAKIEAEIADLEDILAKPERQRGIV 480

Query 481 RDELAEIVEKHGDARRTRIVAADGDVSDEDLIAREDVVVTITETGYAKRTKTDLYRSQKR 540

RDELAEIVEKHGDARRTRIVAADGDVSDEDLIAREDVVVTITETGYAKRTKTDLYRSQKR

Sbjct 481 RDELAEIVEKHGDARRTRIVAADGDVSDEDLIAREDVVVTITETGYAKRTKTDLYRSQKR 540

Query 541 GGKGVQGAGLKQDDIVRHFFVCSTHDWILFFTTQGRVYRAKAYELPEASRTARGQHVANL 600

GGKGVQGAGLKQDDIV HFFVCSTHDWILFFTTQGRVYRAKAYELPEASRTARGQHVANL

Sbjct 541 GGKGVQGAGLKQDDIVGHFFVCSTHDWILFFTTQGRVYRAKAYELPEASRTARGQHVANL 600

Query 601 LAFQPEERIAQVIQIRSYEDAPYLVLATRNGLVKKTKLTDFDSNRSGGIVAINLRDNDEL 660

LAFQPEERIAQVIQIRSYEDAPYLVLATRNGLVKKTKLTDFDSNRSGGIVAINLRDNDEL

Sbjct 601 LAFQPEERIAQVIQIRSYEDAPYLVLATRNGLVKKTKLTDFDSNRSGGIVAINLRDNDEL 660

Query 661 VGAVLCSAEDDLLLVSANGQSIRFSATDEALRPMGRATSGVQGMRFNADDYLLSLNVVRE 720

VGAVLCSAEDDLLLVSANGQSIRFSATDEALRPMGRATSGVQGMRFNADDYLLSLNVVRE

Sbjct 661 VGAVLCSAEDDLLLVSANGQSIRFSATDEALRPMGRATSGVQGMRFNADDYLLSLNVVRE 720

Query 721 GTYLLVATSGGYAKRTAIEEYPVQGRGGKGVLTVMYDRRRGRLVGALIVDEDSELYAITS 780

GTYLLVATSGGYAKRTAIEEYPVQGRGGKGVLTVMYDRRRGRLVGALIVDEDSELYAITS

Sbjct 721 GTYLLVATSGGYAKRTAIEEYPVQGRGGKGVLTVMYDRRRGRLVGALIVDEDSELYAITS 780

Query 781 GGGVIRTAAGQVRKAGRQTKGVRLMNLGEGDTLLAIARNAEEAADEAVDESDGAAGSDG 839

GGGVIRTAAGQVRKAGRQTKGVRLMNLGEGDTLLAIARNAEEAADEAVDESDGAAGSDG

Sbjct 781 GGGVIRTAAGQVRKAGRQTKGVRLMNLGEGDTLLAIARNAEEAADEAVDESDGAAGSDG 839
