# Supplementary material for: Comparative Genomics of Mycobacterium avium Subspecies Paratuberculosis Sheep Strains
Source: Front Vet Sci. 2021 Feb 15;8:637637. doi: 10.3389/fvets.2021.637637 (PMC7917049; doi:10.3389/fvets.2021.637637)
Supplement: Supplementary Material 4 — BLASTx results of lineage-specific genes. [file Data_Sheet_4.docx]

Lineage-specific genes of interest BLASTx top results. Where the top hit was a hypothetical protein but another significant hit to a related species was obtained, both were recorded.

| **Roary** | | | **BLASTx** | | | | | | |
| --- | --- | --- | --- | --- | --- | --- | --- | --- | --- |
| **ID** | **Annotation** | **Type** | **Description** | **Max score** | **total score** | **Query cover** | **E-value** | **Percent identity** | **Accession** |
| group4585 | hypothetical protein | I | [hypothetical protein [Mycobacterium avium]](https://blast.ncbi.nlm.nih.gov/Blast.cgi#alnHdr_WP_023885779) | 100 | 100 | 96% | 9.00E-27 | 90.20% | [WP_023885779.1](https://www.ncbi.nlm.nih.gov/protein/WP_023885779.1?report=genbank&log$=prottop&blast_rank=1&RID=KGS5R2EP014) |
|  |  |  | [nuclear transport factor 2 family protein [Mycobacterium lepraemurium]](https://blast.ncbi.nlm.nih.gov/Blast.cgi#alnHdr_WP_128617474) | 100 | 100 | 96% | 1.00E-26 | 90.20% | [WP_128617474.1](https://www.ncbi.nlm.nih.gov/protein/WP_128617474.1?report=genbank&log$=prottop&blast_rank=3&RID=KGS5R2EP014) |
| group4593 | hypothetical protein | I | [hypothetical protein O976_26305 [Mycobacterium avium subsp. paratuberculosis 10-8425]](https://blast.ncbi.nlm.nih.gov/Blast.cgi#alnHdr_ETB45574) | 204 | 204 | 99% | 3.00E-64 | 99.03% | [ETB45574.1](https://www.ncbi.nlm.nih.gov/protein/ETB45574.1?report=genbank&log$=prottop&blast_rank=1&RID=KGSDBS7G014) |
|  |  |  | [MMPL family transporter [Mycobacterium avium]](https://blast.ncbi.nlm.nih.gov/Blast.cgi#alnHdr_WP_019729156) | 204 | 204 | 99% | 1.00E-63 | 99.03% | [WP_019729156.1](https://www.ncbi.nlm.nih.gov/protein/WP_019729156.1?report=genbank&log$=prottop&blast_rank=3&RID=KGSDBS7G014) |
| cinA1 | 1,8-cineole 2-endo-monooxygenase | I | [cytochrome P450 [Mycobacterium avium]](https://blast.ncbi.nlm.nih.gov/Blast.cgi#alnHdr_WP_019684507) | 904 | 904 | 99% | 0 | 99.78% | [WP_019684507.1](https://www.ncbi.nlm.nih.gov/protein/WP_019684507.1?report=genbank&log$=prottop&blast_rank=1&RID=KGSHF06G014) |
| group4493 | hypothetical protein | I | [hemolysin III family protein [Mycobacterium avium]](https://blast.ncbi.nlm.nih.gov/Blast.cgi#alnHdr_WP_019685052) | 370 | 370 | 99% | 2.00E-130 | 100.00% | [WP_019685052.1](https://www.ncbi.nlm.nih.gov/protein/WP_019685052.1?report=genbank&log$=prottop&blast_rank=1&RID=KGSRE9K3014) |
| mhpA2 | 3-(3-hydroxy-phenyl)propionate/3-hydroxycinnamic acid hydroxylase | I | [FAD-dependent monooxygenase [Mycobacterium avium]](https://blast.ncbi.nlm.nih.gov/Blast.cgi#alnHdr_WP_019730100) | 327 | 460 | 99% | 5.00E-121 | 99.44% | [WP_019730100.1](https://www.ncbi.nlm.nih.gov/protein/WP_019730100.1?report=genbank&log$=prottop&blast_rank=1&RID=KGSVAMRU014) |
| group4592 | hypothetical protein | I | [MMPL family protein [Mycobacterium avium MAV_061107_1842]](https://blast.ncbi.nlm.nih.gov/Blast.cgi#alnHdr_ETZ43695) | 266 | 266 | 99% | 3.00E-87 | 98.82% | [ETZ43695.1](https://www.ncbi.nlm.nih.gov/protein/ETZ43695.1?report=genbank&log$=prottop&blast_rank=1&RID=KGSXRFER014) |
| group1815 | hypothetical protein | I | [hypothetical protein [Mycobacterium avium]](https://blast.ncbi.nlm.nih.gov/Blast.cgi#alnHdr_WP_003874661) | 264 | 321 | 99% | 5.00E-87 | 98.47% | [WP_003874661.1](https://www.ncbi.nlm.nih.gov/protein/WP_003874661.1?report=genbank&log$=prottop&blast_rank=1&RID=KGT307E8014) |
| group4617 | hypothetical protein | I | [TetR/AcrR family transcriptional regulator [Mycobacterium avium]](https://blast.ncbi.nlm.nih.gov/Blast.cgi#alnHdr_WP_003876512) | 371 | 371 | 92% | 3.00E-130 | 94.42% | [WP_003876512.1](https://www.ncbi.nlm.nih.gov/protein/WP_003876512.1?report=genbank&log$=prottop&blast_rank=1&RID=KGTBJ85T016) |
| group4500 | hypothetical protein | I | [MULTISPECIES: nitroreductase family protein [Mycobacterium avium complex (MAC)]](https://blast.ncbi.nlm.nih.gov/Blast.cgi#alnHdr_WP_003873585) | 240 | 299 | 92% | 1.00E-79 | 91.18% | [WP_003873585.1](https://www.ncbi.nlm.nih.gov/protein/WP_003873585.1?report=genbank&log$=prottop&blast_rank=1&RID=KGTJD7DZ016) |
| group4778 | hypothetical protein | III | [TetR/AcrR family transcriptional regulator [Mycobacterium avium]](https://blast.ncbi.nlm.nih.gov/Blast.cgi#alnHdr_WP_003876512) | 400 | 400 | 93% | 1.00E-141 | 100.00% | [WP_003876512.1](https://www.ncbi.nlm.nih.gov/protein/WP_003876512.1?report=genbank&log$=prottop&blast_rank=1&RID=KGSKR4K2014) |
| group4772 | hypothetical protein | III | [MULTISPECIES: nitroreductase family protein [Mycobacterium avium complex (MAC)]](https://blast.ncbi.nlm.nih.gov/Blast.cgi#alnHdr_WP_003873585) | 286 | 286 | 99% | 4.00E-98 | 100.00% | [WP_003873585.1](https://www.ncbi.nlm.nih.gov/protein/WP_003873585.1?report=genbank&log$=prottop&blast_rank=1&RID=KGTG9D2D014) |
| group4781 | hypothetical protein | III | [hypothetical protein MAPs_43080 [Mycobacterium avium subsp. paratuberculosis S397]](https://blast.ncbi.nlm.nih.gov/Blast.cgi#alnHdr_EGO39109) | 517 | 517 | 99% | 0 | 100.00% | [EGO39109.1](https://www.ncbi.nlm.nih.gov/protein/EGO39109.1?report=genbank&log$=prottop&blast_rank=1&RID=KGS1JJE5014) |
| group4363 | hypothetical protein | III | [MULTISPECIES: hemolysin III family protein [Mycobacterium avium complex (MAC)]](https://blast.ncbi.nlm.nih.gov/Blast.cgi#alnHdr_WP_003875366) | 459 | 459 | 99% | 1.00E-163 | 100.00% | [WP_003875366.1](https://www.ncbi.nlm.nih.gov/protein/WP_003875366.1?report=genbank&log$=prottop&blast_rank=1&RID=KGRTZ4RS014) |
