# Supplementary material for: Comparative Genomics of Mycobacterium avium Subspecies Paratuberculosis Sheep Strains
Source: Front Vet Sci. 2021 Feb 15;8:637637. doi: 10.3389/fvets.2021.637637 (PMC7917049; doi:10.3389/fvets.2021.637637)
Supplement: Supplementary Material 5 — Previously identified variable loci from Castellanos et al. (43) in sheep MAP strains. [file Data_Sheet_5.docx]

Supplementary material. 5 Previously reported variable regions in MAP Type I and Type III by microarray analysis with K10. Where these genes were also found as lineage-specific in the present study, gene IDs have been provided.

| **Locus ID (Previous study)** | **Gene ID (Present study)** | **Castellanos et al. 2009 findings** | **Comments** |
| --- | --- | --- | --- |
| MAP2704 (haemolysin III like) | Group 4493 (Type I)  Group 4363 (Type III) | Deletions in Type I isolates | Supported, Type I isolates have a 427bp deletion |
| MAP3460c (transposase) | Not found by present study method as lineage-specific | Deletions in Type I isolates | Highly variable within Type I and III |
| MAP1433c to MAP1438c (lipid metabolism) | Not found by present study as lineage-specific | Deletions in Type III isolates | Mostly supported, exceptions in isolates  SRR3050018 and JIII386, hence was not identified by the method used in the present study. Some synonymous SNPs in Type I isolates |
| MAP3584 (alkanesulfonate monooxygenase) | *cinA*1 (Type I) | Deletions in Type III isolates | Mostly supported, deletions in type III isolates were inconsistent with differences in isolates SRR3050018 and JIII386, hence no Type III specific protein was identified by the method used in the present study.  Identical in all Type I isolates and K10 |
| MAP2325 | Not found by present study as lineage-specific | Previously reported as absent from Australian isolates. All tested strains contained this gene | Absent in all Australian isolates, present in Type I isolates from New Zealand and other countries, some SNPs present in Type III isolates |

Supplementary table. 2 Lineage-specific genes and variation of type I and type III isolates and their differences to the K10 (Type II) reference genome. Where Prokka annotated the gene as a hypothetical protein but BLASTx was able to provide a putative annotation, the BLASTx annotation was used. Variations are from BLASTp results.

| **Gene** | **Type** | **Annotation** | **Variations*** | **K10 variations** |
| --- | --- | --- | --- | --- |
| Group 4585 | I | Putative nuclear transport factor 2 family protein | 5 variable mismatches in the Type III protein | 5 mismatches to and one amino acid shorter than the Type I lineage protein |
| Group 4593 | I | MMPL family transporter | No significant protein hit in Type III isolates | No significant hit to this protein in the K10 genome |
| *cinA*1 | I | 1,8-cineole 2-endo-monooxygenase | Type III protein has 8-17 mismatches, length is identical between lineages | Protein is 41 amino acids shorter and has three mismatches to the Type I lineage protein |
| *mhpA*2 | I | 3-(3-hydroxy-phenyl)propionate/3-hydroxycinnamic acid hydroxylase | Type I isolate protein sequences are 62 amino acids longer and Type III isolates have a single mismatch | K10 has a significant hit that is 42 amino acids shorter with two mismatches |
| Group 4493 | I | Hemolysin III family protein | Type III protein is 54 amino acids longer and contains 9 mismatches to the Type I version | 54 amino acids shorter in K10 with 10 mismatches |
| Group 4363 | III |  |  | Single mismatch to Type III protein |
| Group 4592 | I | MMPL family protein | No significant protein hit in Type III isolates | No significant hit to this protein in the K10 genome |
| Group 1815 | I | Hypothetical protein | 1-2 mismatches in Type III protein and Type III is 22 amino acids shorter | 22 amino acids shorter and two mismatches in K10 |
| Group 4617 | I | *TetR/AcrR* family transcriptional regulator | 11 amino acid mismatches and the Type I protein is three amino acids shorter | K10 protein is identical to Type III version |
| Group 4778 | III |  |  |  |
| Group 4500 | I | Nitroreducatase family protein | Type I protein is 185 amino acids long and Type III is 171. Contains 11 mismatches | K10 protein is 15 amino acids shorter and contains 11 mismatches to the Type I version |
| Group 4772 | III |  |  | K10 protein is identical to Type III version |
| Group 4781 | III | Hypothetical protein | Type I isolates 37-164 amino acids long with 24-105 mismatches. Only seven of these hits in Type I isolates are significant (e-value < 0.05). Type III are all 299 amino acids long with a single mismatch present in four isolates | No significant hit to this protein in the K10 genome |

*bp = base pairs
